# Determinants of out-of-hospital cardiac arrest (OHCA) and associated outcomes at a single center in Dubai

**DOI:** 10.1186/s12245-026-01174-5

**Published:** 2026-03-13

**Authors:** Farheen Memon, Salman Muhammad Soomar, Desh Deepak, Mariam Alqemzi, Layanah Matarneh, Amal Almheiri, Mohammed Abdul Raheem

**Affiliations:** 1 King’s College Hospital London, Dubai, UAE; 2 Mohammed Bin Rashid University of Medicine and Health Sciences, Dubai, UAE

**Keywords:** Out-of-hospital cardiac arrest, Cardiopulmonary resuscitation, Survival rate

## Abstract

**Background:**

Out-of-hospital cardiac arrest is a global health challenge with survival rates influenced by timely intervention and regional healthcare dynamics. Despite extensive research in Western contexts, data from rapidly urbanizing regions, such as Dubai, are scarce. This study aims to determine the survival and outcomes of Out-of-hospital cardiac arrest patients visiting emergency department.

**Methods:**

This is a single-center, small observational study which used electronic health records and emergency logs to identify 48 Out-of-hospital cardiac arrest cases from a single healthcare center (January 2020–October 2024). Out-of-hospital cardiac arrest cases with resuscitation attempts and complete records were included only. Cox proportional hazard analysis was applied to determine the association between survival predictors and Out-of-hospital cardiac arrest outcomes.

**Results:**

The majority were male (93.75%), belonging to low socioeconomic status (83.33%). Bystanders were present in 75% of cases, but only 22.92% performed cardiopulmonary resuscitation. Mean emergency medical services response time was 15.75 ± 8.55 min. Multivariable analysis identified the absence of bystanders (HR = 3.58, 95% CI1.53–5.51), no bystander cardiopulmonary resuscitation (HR = 2.12, 95% CI1.82–4.58), prior cardiac arrest (HR = 7.35, 95% CI1.81–9.76), and lack of ROSC (HR = 5.99, 95% CI2.16–8.38) as significant mortality predictors.

**Conclusion:**

The survival rate in this study (20.83%) reflects intermediate outcomes, constrained by low bystander cardiopulmonary resuscitation rates and prolonged EMS response times. Outcomes could be improved via targeted public cardiopulmonary resuscitation training, automated external defibrillator, accessibility, and optimized emergency response systems.

**Clinical trial number:**

Not applicable.

## Introduction

Out-of-hospital cardiac arrest (OHCA) represents a dire and time-sensitive medical emergency that poses a formidable challenge to healthcare systems worldwide. It affects 96 people out of 100,000 worldwide [1].

It is an emergency condition characterized by the sudden cessation of cardiac activity outside the confines of a clinical setting, often striking individuals within their homes, workplaces, or public spaces [2]. OHCA is a life-threatening event where a person’s heart suddenly stops beating, and it typically occurs in non-medical settings like homes and public places [3]. The outcome of OHCA can vary widely and depends on factors such as timeliness, quality of response, and advancements in medical technology. OHCA survival rates vary based on factors like location, bystander intervention, and Emergency Medical Services (EMS) response time [4]. Early initiation of cardiopulmonary resuscitation (CPR) and rapid defibrillation significantly improve survival chances. High-quality CPR, involving effective chest compressions and minimal interruptions, is critical. Techniques like high-performance CPR and feedback devices are explored to enhance resuscitation efforts [5]. OHCA is an event of profound consequence, as it can result in devastating consequences for the affected individuals, their families, and society at large. Survival from OHCA hinges on a complex interplay of factors, including rapid access to appropriate care, effective CPR, defibrillation, and post-resuscitation care within a conducive healthcare system [6]. While the global burden of OHCA is well-documented, the landscape of OHCA in specific regions and cities is shaped by unique demographic, cultural, and healthcare characteristics [7]. While numerous studies have explored OHCAs in various contexts, there remains a notable gap in our understanding of how these incidents manifest in urban environments with diverse populations. Urban settings often exhibit distinct characteristics, such as varying healthcare infrastructure, population density, and response times, which can significantly influence the dynamics of OHCAs [8]. OHCA affects diverse demographics, with higher incidences among older adults (65+), men, and those with pre-existing cardiovascular conditions [9]. Socioeconomic factors contribute, with lower-income communities facing potential barriers to timely emergency services. Rural areas grapple with longer response times and limited healthcare access, while urban areas contend with higher population density and stressors [8]. Disparities in healthcare and education further influence outcomes [10]. Recognizing these factors is essential for targeted interventions and improving OHCA outcomes across varied populations.

Understanding the patient demographics and clinical features of OHCA in Dubai is of paramount importance. Studying the demographics of OHCA patients is essential for refining emergency medical services. By identifying high-risk areas and understanding demographic trends, response times can be optimized, targeted public awareness campaigns can be developed, and training programs can be tailored to specific populations. Strategic placement of automated external defibrillators (AEDs), community engagement efforts, and research initiatives based on demographic data all contribute to an improved and more responsive OHCA management system, ultimately enhancing outcomes and saving lives.

This research endeavors to provide a comprehensive exploration of OHCA in Dubai by conducting a retrospective observational study in a single center, delving into patient characteristics, clinical patterns, and outcomes. This study aims to determine the survival and outcomes of OHCA patients visiting emergency department of King’s College Hospital London, Dubai.

## Operational definitions

1. “Complete Recovery” was considered in this study when the person who survived the event and returned to their previous level of physical, mental, and neurological functioning without significant long-term damage [11].
2. “Complete Neurological Recovery” was considered when Patients have recovered completely if they were released with CPC 1 (good cerebral performance) or CPC 2 (moderate handicap but independent in activities of daily living). CPC status was determined based on medical record documentation at hospital release, which included degree of consciousness, cognitive function, ability to communicate, mobility, and the need for institutional or long-term support care [12].
3. “Survival Time” was the period between ROSC achieved to death.
4. Patients were censored at the time of Emergency Department (ED) or hospital discharge and the conclusion of the 30-day follow-up period. Patients who were lost to follow-up before 30 days were filtered based on their last known alive date.
5. A case was classified as having incomplete records if one or more of the following essential variables were missing or undocumented in the electronic health record: survival status at hospital discharge, ROSC status, EMS response time, initial cardiac rhythm and documentation of resuscitation attempt.
6. Do-Not-Attempt-Resuscitation (DNAR) defined as patients with a documented physician-signed order or legally recognized directive indicating that no cardiopulmonary resuscitation (CPR) should be initiated in the event of cardiac arrest.

## Methods

### Study design

The study employs a retrospective observational design to analyze OHCA cases in a single center in Dubai.

### Study setting

The study is conducted at King’s College Hospital London, Dubai, UAE. The center serves as the primary location for data collection, analysis, and investigation of OHCA cases.

### Study duration

The study was conducted using data between January 1, 2020, and October 31, 2024.

### Study population

All adult patients suffered cardiac arrest out of the hospital.

### Eligibility criteria

This study included adult patients (≥ 18 years) who experienced out-of-hospital cardiac arrest (OHCA) and were transported to King’s College Hospital London, Dubai, between January 1, 2020, and October 31, 2024, with resuscitation attempts initiated. Pediatric patients (< 18 years), documented DNAR cases identified prior to data abstraction, pregnancy-related arrests, non-residents of Dubai, and cases with incomplete medical documentation were excluded.

### Case identification transparency

During the study period, a total of 119 potential OHCA cases were identified through electronic health records and emergency logs. Of these, 21 were excluded due to due to DNAR status, 24 due to incomplete records (outcome status, ROSC documentation and EMS response time), and 26 due to non-residency. The final analytic cohort consisted of 48 adult OHCA cases.

### Sampling technique and sample size

No statistical sampling was performed. All eligible adult OHCA cases meeting inclusion criteria during the study period were consecutively included. Of 119 potential cases identified, exclusions were applied for DNAR status, incomplete records, and non-residency. A sample of 48 OHCA cases was included in the study using a non-probability purposive sampling technique.

### Data collection procedure

The identification phase involved compiling OHCA cases within the study center during the designated study period using electronic health records (EHR) and emergency response logs. The pre-defined inclusion and exclusion criteria were used to filter eligible cases based on location, time frame, age, gender, clinical setting, documentation, and resuscitation status. Comprehensive medical records, encompassing demographic information and clinical data, were systematically retrieved for each identified case, emphasizing the maintenance of consistency and accuracy in data variables. Standardized data abstraction forms facilitated the organized collection of key information such as age, gender, socioeconomic status, time and location of cardiac arrest, response time, resuscitation measures, and outcomes. Quality control measures were implemented to ensure accuracy, and the abstracted data were securely entered into electronic databases. Strict adherence to confidentiality and ethical considerations was maintained throughout the process, and data security was prioritized. Verification and validation were conducted to confirm the accuracy and reliability of the entered data.

The primary outcome was survival to hospital discharge. Neurological outcome at discharge was assessed using the Cerebral Performance Category (CPC) scale.

### Clinical methodological details

All vascular access procedures, including peripheral intravenous (IV) and central venous catheterization, were carried out in accordance with a standardized, evidence-based institutional policy that followed AHA recommendations. Regular staff training in the emergency room, the use of procedure checklists, and continuing quality audits incorporated into the hospital’s clinical governance framework all helped to reinforce protocol compliance.

### Handling of missing data

Missing data were analyzed for completeness and trend. Complete-case analysis was applied to variables with less than 5% missing data. We performed sensitivity analyses on variables with increased missingness, but no imputation methods were used because the fraction of missing data was minor and unlikely to alter the study conclusions. We provided these details to increase transparency in data processing.

### Statistical analysis

Given the limited sample size, analyses were primarily descriptive. Continuous variables which were normally distributed were summarized using mean ± standard deviation and skewed variables were summarized as median (interquartile range) as appropriate. Shapiro-wilk test was used to check normal distribution. Categorical variables were expressed as frequencies and percentages. Between-group comparisons were performed using independent sample t-tests or Fisher’s exact tests where appropriate. Exploratory time-to-event analysis was conducted using Kaplan–Meier methods. Cox proportional hazards regression was performed as an exploratory multivariable analysis using variables bystander presence, bystander CPR, prior cardiac arrest, ROSC achieved. P-values ≤ 0.05 were considered significant. All the analysis was carried on R software.

## Results

Of the 48 patients, the majority were male 45 (93.75%). The mean ± standard deviation (SD) age was 45.72 ± 12.10. More than 80% of the participants were Asian. Out of 48 patients, 40 (83.33%) had low socioeconomic status and most had no comorbidities. Majority of arrests were witnessed by bystanders and were present in 36 (75.00%) of the cases. However, CPR by a bystander was given to only 11 (22.92%) patients. EMS response time was (mean ± SD) 15.75 ± 8.55. Shock was delivered to 14 (29.17%) of patients. The majority had the Unshockable initial rhythm 34 (70.83%). Bag mask ventilation was the most common airway management strategy utilized 26 (54.16%). As per the standard American Heart Association (AHA) 1 mg (median) Epinephrine was given every 3–5 min. The survival to discharge was 20.83% (*n* = 10) and favorable neurological recovery was 90% (*n* = 9/10).

Only 1 (2.08%) had a previous history of cardiac arrest. Return of spontaneous circulation (ROSC) was achieved in 19 (39.58%) of the patients (Table 1).

**Table 1:** Baseline and clinical characteristics of cardiac arrest in OHCA patients (*n* = 48)

Characteristics	*n* (%)
Age (mean ± SD)	45.72 ± 12.10
Gender	
Male	45 (93.75)
Female	3 (6.25)
Employment	
Unemployed	6 (12.50)
Employed	28 (58.33)
Unknown	14 (29.17)
Nationality	
Emiratis	2 (4.17)
Indian	25 (52.08)
Pakistani	10 (20.83)
Sri Lankan	2 (4.17)
Filipino	3 (6.25)
Bangladeshi	2 (4.17)
Others	4 (8.33)
Low socio-economic status	
No	5 (10.42)
Yes	40 (83.33)
Unknown	3 (6.25)
Comorbidities	
No	38 (79.16)
Hypertension	4 (8.33)
Diabetes	2 (4.17)
Cerebellar hemorrhage	1 (2.08)
Chronic kidney disease	1 (2.08)
Dementia	1 (2.08)
COVID-19	1 (2.08)
Witness status	
Bystander	36 (75.00)
Unwitnessed	12 (25.00)
Bystander present	
No	12 (25.00)
Yes	36 (75.00)
Bystander CPR	
No	37 (77.08)
Yes	11 (22.92)
EMS response time (mean ± SD)	15.75 ± 8.55
Initial rhythm/ first documented rhythm	
Unshockable	34 (70.83)
Shockable	14 (29.17)
Shock delivered	
No	34 (70.83)
Yes	14 (29.17)
Airway management strategy	
Bag mask ventilation	26 (54.16)
Supraglottic Airway	14 (29.17)
Endotracheal intubation	8 (16.67)
Epinephrine mg (Median, IQR)	1.0 (1.0–3.0)
Prior CVS conditions	
No	40 (83.33)
Yes	8 (16.67)
Previous cardiac arrest	
No	47 (97.92)
Yes	1 (2.08)
ROSC achieved	
No	29 (60.42)
Yes	19 (39.58)

Out of 48, 10 (20.83%) of the patients were alive. Out of those 10, 9 (90.00%) were completely recovered (Table 2).

**Table 2:** Survival characteristics of OHCA patients (*n* = 48)

**Outcome**	
Deceased	38 (79.17)
Alive	10 (20.83)
Neurological status at discharge (*n* = 10)	
Complete recovery	9 (90.00)
Brain Injury	1 (10.00)

The age was almost the same for both dead and alive patients [45.63 ± 12.23 & 46.1 ± 12.18]. The majority in both dead and alive groups were males [37 (97.37%) & 8 (80.00%)] and from low socio-economic status. Those who died didn’t have a history of previous cardiac arrest [38 (100.00%)] (Table 3).

**Table 3:** Baseline and clinical characteristics stratified on outcome of patients (*n* = 48)

Characteristics	Outcome		*P*-value
	Dead (*n* = 38)	Alive (*n* = 10)	
Age (mean ± SD)	45.63 ± 12.23	46.1 ± 12.18	0.202***
Gender			0.044**
Male	37 (97.37)	8 (80.00)	
Female	1 (2.63)	2 (20.00)	
Low socio-economic status			0.434**
No	3 (7.89)	2 (20.00)	
Yes	33 (86.84)	7 (70.00)	
Unknown	2 (5.26)	1 (10.00)	
Comorbidities			0.207*
No	30 (78.95)	8 (80.00)	
Hypertension	4 (10.53)	0 (0.00)	
Diabetes	2 (5.26)	0 (0.00)	
Cerebellar hemorrhage	1 (2.63)	0 (0.00)	
Chronic kidney disease	0 (0.00)	1 (10.00)	
Dementia	0 (0.00)	1 (10.00)	
COVID-19	1 (2.63)	0 (0.00)	
Bystander present			0.004*
No	6 (15.79)	6 (60.00)	
Yes	32 (84.21)	4 (40.00)	
Bystander CPR			0.275*
No	28 (73.68)	9 (90.00)	
Yes	10 (26.32)	1 (10.00)	
EMS response time (mean ± SD)	16.25 ± 13.14	15.68 ± 7.98	0.904***
Initial rhythm			0.474*
Unshockable	26 (68.42)	8 (80.00)	
Shockable	12 (31.58)	2 (20.00)	
Prior CVS conditions			0.204*
No	33 (86.84)	7 (70.00)	
Yes	5 (13.16)	3 (30.00)	
Previous cardiac arrest			0.049*
No	38 (100.00)	9 (90.00)	
Yes	0 (0.00)	1 (10.00)	
ROSC achieved.			< 0.001*
No	29 (76.32)	0 (0.00)	
Yes	9 (23.68)	10 (100.00)	

*Chi-square **Fisher’s exact test ***ANOVA

**Fig. 1:**
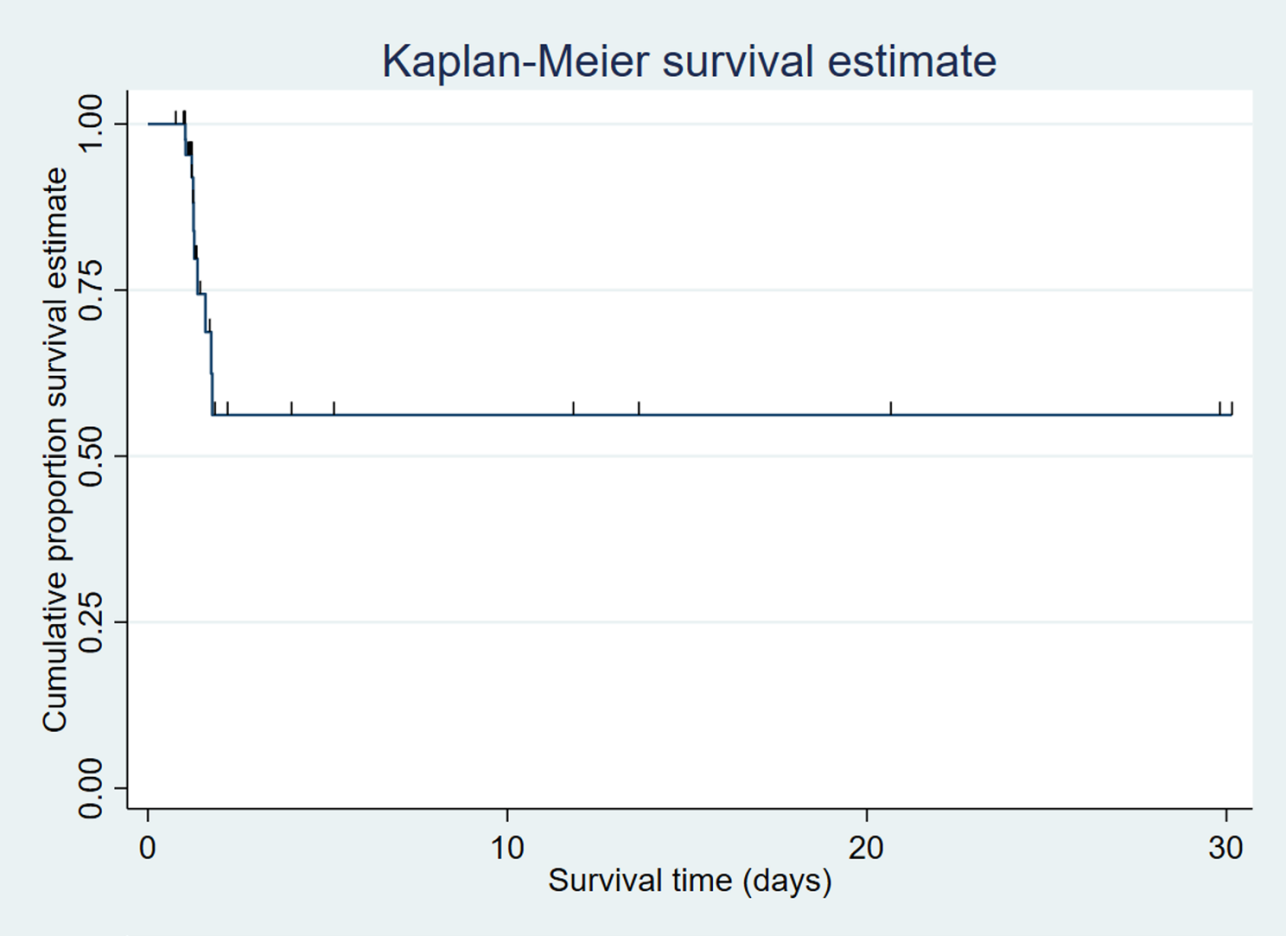
Graph presenting OHCA patient’s overall survival in days (*n* = 48)

The median (Intra Quartile Range) survival time (in days) was 0.40 (0.21–11.76) (Fig. 1).

Multivariable Cox proportion showed that “no bystander present, no bystander CPR, previous history of cardiac arrest, and no ROSC achieved” were significant risk factors for the survival of patients in OHCA. Multivariable Cox proportional hazards regression modeling time to death demonstrated that unwitnessed arrest was associated with a significantly higher hazard of mortality (HR 3.58, 95% CI 1.53–5.51) compared with witnessed arrest. Absence of bystander CPR was also independently associated with increased mortality risk (HR 2.12, 95% CI 1.82–4.58). A prior history of cardiac arrest was associated with higher mortality (HR 7.35, 95% CI 1.81–9.76), and failure to achieve ROSC was strongly associated with death (HR 5.99, 95% CI 2.16–8.38). These hazard ratios represent relative mortality risk rather than probability of survival (Table 4).

**Table 4:** Multivariable survival crude and adjusted hazard ratios (*n* = 48)

Characteristic	Crude HR (95% CI)	*p*-value	Adjusted HR (95% CI)	*p*-value
Age (years)			-	-
21–30	1		-	-
31–40	0.39 (0.33–2.84)		-	-
41–50	0.21 (0.13–1.56)	0.668	-	-
51–60	0.31 (0.22–2.22)		-	-
> 60	0.47 (0.36–3.49)		-	-
Gender				
Female	1	0.144*	1	
Male	3.85 (1.38–8.05)		2.47 (0.72–6.82)	0.097
Low socio-economic status			-	-
No	1	0.483	-	-
Yes	1.19 (0.61–2.33)		-	-
Comorbidities			-	-
No Hypertension Diabetes Cerebellar hemorrhage Chronic kidney disease Dementia COVID-19	1 1.19 (0.50–2.69) 1.36 (0.67–3.50) 1.51 (0.76–2.47) 1.46 (1.60–2.69) 1.33 (0.65–4.90) 0.41 (0.35–2.16) 0.76 (0.48–3.53)	0.692	-	-
Bystanders present				
Yes	1		1	
No	3.58 (1.53–5.51)	0.008*	3.58 (1.53–5.51)	0.009**
Bystander CPR				
Yes	1		1	
No	3.76 (1.47–6.20)	0.039*	2.12 (1.82–4.58)	0.027**
EMS response time				
0–10 min	1		1	
11–20 min	1.27 (1.14–3.32)		1.05 (0.87–2.68)	
21–30 min	1.40 (1.26–3.83)	0.176*	2.88 (0.94–5.31)	0.453
> 30 min	6.14 (2.29–9.73)		3.94 (1.21–6.84)	
Initial rhythm			-	-
Unshockable	1		-	-
Shockable	1.91 (0.40–3.75)	0.387	-	-
Prior CVS conditions			-	-
No	1		-	-
Yes	2.19 (0.56–5.82)	0.283	-	-
Previous cardiac arrest				
No	1		1	
Yes	2.08 (1.13–5.91)	0.003*	7.35 (1.81–9.76)	0.004**
ROSC achieved.				
Yes	1		1	
No	1.34 (1.19–3.88)	< 0.001*	5.99 (2.16–8.38)	< 0.001**

*Significant at Univariate **Significant at Multivariable

## Discussion

In this study 48 cases of OHCA were included. Most patients were male (93.75%), with a mean age of 45.72 ± 12.10 years, and more than 80% of the subjects were of Asian descent and belonged to a low socioeconomic status. Although 75% OHCA were witnessed but only 22.92% received CPR through bystanders. The mean emergency medical services response time was 15.75 ± 8.55 min. Most patients (70.83%) had an unshockable initial rhythm. Return of spontaneous circulation was achieved in 39.58% of cases. The survival rate of patients was recorded at 20.83% at the time of discharge from the hospital. Almost 90% of the survivors had complete neurological recovery. Significant factors associated with survival of patients were bystander presence, bystander CPR, previous cardiac arrest history, and ROSC achievement. The results of the study show that immediate intervention and emergency response efficiency have a significant impact on OHCA outcomes.

The survival rate in this study (20.83%) appears slightly higher than reported in some regions but remains lower than in high-income countries with optimized emergency response systems. Studies from high-income countries in North America and Europe report OHCA survival rates ranging from 10% to 25%. The outcomes of these studies depend on variables such as bystander CPR rates, EMS efficiency, and AED availability [13, 14]. Low and middle-income countries (LMICs) had survival rates as low as 5–15%, mainly due to delayed EMS response and lower bystander CPR rates, according to a study published by Matsuyama T et al. (2020) [15].

A study by Kiyohara K et al. (2022) found that regions with high public awareness and CPR training had significantly better OHCA survival rates, with a record bystander CPR rate exceeding 50% in Japan [16]. In this study bystander CPR rate was only 22.92% compared to Japan, which indicates a need for improved public resuscitation training programs for emergencies of this nature. Low EMS response time, under 10 min, is significantly correlated with higher survival rates [17]. This could be a major limiting factor in improving survival outcomes when compared to the mean EMS response time in this study, which was 15.75 min.

The proportion of unshockable initial rhythms in this study was high at 70.83%, which is consistent with the results of previous studies found by Sawyer et al. (2022) in their review. They found that in Asia and the Middle East, cardiovascular risk factors and limited interventions before hospital admissions contributed to poor initial cardiac rhythms [18]. The common use of AEDs and advanced resuscitation public training in developed countries has higher rates of shockable rhythms, which lead to improved survival outcomes.

There are several factors responsible for OHCA outcomes in this study when compared to other regions. One major factor is the demographic composition of OHCA patients. More than 80% of OHCA cases involved Asian expatriates in Dubai, belonging to a lower socioeconomic background, and this is a trend that is uncommon in Western and European countries, where OHCA data typically shows a more ethnically homogeneous population [19]. This demographic difference might have influenced survival rates due to differences in behavior, access to healthcare, and socioeconomic factors.

Bystander CPR rates in this study (22.92%) were found to be significantly lower compared to developed countries, including Sweden (70%) and the United States of America (USA) (50–60%) [20]. That shows a major gap in public CPR training programs in the region. Literature mentioned that in cities with high OHCA survival rates, widespread CPR education, AED accessibility, and dispatcher-assisted CPR can significantly improve OHCA outcomes [8]. Implementation of similar public health initiatives can enhance OHCA survival rates in Dubai as EMS response time in this study was 15.75 min, which is significantly longer compared to the benchmark set in high-performance emergency medical systems in countries such as Poland, where the average response time was 7.92 min, according to a 2024 study by Goniewicz M et al., [21]. Optimized ambulance dispatch, traffic management, and pre-hospital triaging systems can significantly reduce response times, which could improve OHCA survival. The overall ROSC achievement rate in this study was 39.58%, which is comparable to international benchmarks. This suggests that once resuscitation is initiated, in-hospital care quality meets global standards. However, to improve survival rates in the community, engagement and CPR training for the public are recommended.

## Limitations

This study has several important limitations. First, the single-center retrospective design limits generalizability and introduces potential selection bias. Second, the small sample size (*n* = 48) substantially limits statistical power and restricts robust multivariable modeling. Third, only patients transported to a tertiary private hospital were included, potentially introducing survivorship and referral bias. Fourth, EMS system-level variables, dispatcher-assisted CPR data, and detailed prehospital termination decisions were unavailable. Fifth, documentation-based comorbidity data may underestimate true disease burden. Finally, the extreme male predominance likely reflects Dubai’s expatriate labor demographics rather than biological differences, limiting extrapolation to other populations.

## Conclusion

In this single-center exploratory analysis, survival to discharge following OHCA was 20.83%. Bystander presence, CPR, and ROSC were associated with outcomes. Outcomes could be improved via targeted public CPR training, AED accessibility, and optimized emergency response systems. Larger, system-wide registry-based studies are needed to more definitively characterize OHCA epidemiology in Dubai.

## Data Availability

The datasets used and/or analyzed during the current study available from the corresponding author on reasonable request.

## Abbreviations

AED: Automated External Defibrillator
EHR: Electronic Health Records
EMS: Emergency Medical Services
OHCA: Out-of-hospital cardiac arrest
ROSC: Return of Spontaneous Circulation

## References

[1] Porzer M, Mrazkova E, Homza M, Janout V. Out-of-hospital cardiac arrest. Biomed Pap Med Fac Univ Palacky Olomouc Czech Repub. 2017;161(4):348–53. 29235577 10.5507/bp.2017.054

[2] Ong J, O’Connell F, Mazer-Amirshahi M, Pourmand A. An international perspective of out-of-hospital cardiac arrest and cardiopulmonary resuscitation during the COVID-19 pandemic. Am J Emerg Med. 2021;47:192–7. 33894661 10.1016/j.ajem.2021.04.033 PMC8057692

[3] Brooks SC, Clegg GR, Bray J, Deakin CD, Perkins GD, Ringh M, et al. Optimizing outcomes after out-of-hospital cardiac arrest with innovative approaches to public-access defibrillation: a scientific statement from the International Liaison Committee on Resuscitation. Circulation. 2022;145(13):e776–801. 35164535 10.1161/CIR.0000000000001013

[4] Garcia RA, Girotra S, Jones PG, McNally B, Spertus JA, Chan PS, et al. Variation in out-of-hospital cardiac arrest survival across emergency medical service agencies. Circulation: Cardiovasc Qual Outcomes. 2022;15(6):e008755. 10.1161/CIRCOUTCOMES.121.008755 PMC9233095 35698973

[5] Gugelmin-Almeida D, Tobase L, Polastri TF, Peres HHC, Timerman S. Do automated real-time feedback devices improve CPR quality? A systematic review of literature. Resusc plus. 2021;6:100108. 34223369 10.1016/j.resplu.2021.100108 PMC8244494

[6] Rhee BY, Kim B, Lee YH. Effects of prehospital factors on survival of out-of-hospital cardiac arrest patients: age-dependent patterns. Int J Environ Res Public Health. 2020;17(15):5481. 32751367 10.3390/ijerph17155481 PMC7432520

[7] Ho AFW, Lim MJR, Earnest A, Blewer A, Graves N, Yeo JW, et al. Long term survival and disease burden from out-of-hospital cardiac arrest in Singapore: a population-based cohort study. Lancet Reg Health–Western Pac. 2023;32. 10.1016/j.lanwpc.2022.100672 PMC9918801 36785853

[8] Connolly MS, Goldstein JP, Currie M, Carter AJ, Doucette SP, Giddens K, et al. Urban-rural differences in cardiac arrest outcomes: a retrospective population-based cohort study. CJC open. 2022;4(4):383–9. 35495857 10.1016/j.cjco.2021.12.010 PMC9039571

[9] Lai PH, Lancet EA, Weiden MD, Webber MP, Zeig-Owens R, Hall CB, et al. Characteristics associated with out-of-hospital cardiac arrests and resuscitations during the novel coronavirus disease 2019 pandemic in New York City. JAMA Cardiol. 2020;5(10):1154–63. 32558876 10.1001/jamacardio.2020.2488 PMC7305567

[10] Zajacova A, Lawrence EM. The relationship between education and health: reducing disparities through a contextual approach. Annu Rev Public Health. 2018;39(1):273–89. 29328865 10.1146/annurev-publhealth-031816-044628 PMC5880718

[11] Lim C, Verfaellie M, Schnyer D, Lafleche G, Alexander MP. Recovery, long-term cognitive outcome and quality of life following out-of-hospital cardiac arrest. J Rehabil Med. 2014;46(7):691. 24849762 10.2340/16501977-1816 PMC4111096

[12] Dillenbeck E, Svensson L, Rawshani A, Hollenberg J, Ringh M, Claesson A, et al. Neurologic Recovery at Discharge and Long-Term Survival After Cardiac Arrest. JAMA Netw Open. 2024;7(10):e2439196. 39392629 10.1001/jamanetworkopen.2024.39196 PMC11581594

[13] Grubic N, Hill B, Allan KS, Dainty KN, Johri AM, Brooks SC. Community interventions for out-of-hospital cardiac arrest in resource-limited settings: a scoping review across low, middle, and high-income countries. Prehospital Emerg Care. 2023;27(8):1088–100. 10.1080/10903127.2023.2231559 37406163

[14] Blewer AL, Ho AFW, Shahidah N, White AE, Pek PP, Ng YY, et al. Impact of bystander-focused public health interventions on cardiopulmonary resuscitation and survival: a cohort study. Lancet Public Health. 2020;5(8):e428–36. 32768435 10.1016/S2468-2667(20)30140-7

[15] Matsuyama T, Irisawa T, Yamada T, Hayakawa K, Yoshiya K, Noguchi K, et al. Impact of low-flow duration on favorable neurological outcomes of extracorporeal cardiopulmonary resuscitation after out-of-hospital cardiac arrest: a multicenter prospective study. Circulation. 2020;141(12):1031–3. 32202935 10.1161/CIRCULATIONAHA.119.044285

[16] Kiyohara K, Ayusawa M, Nitta M, Sudo T, Iwami T, Nakata K, et al. Characteristics and outcomes of out-of-hospital cardiac arrest among students under school supervision in Japan: a descriptive epidemiological study (2008–2021). Environ Health Prev Med. 2025;30:4. 39805594 10.1265/ehpm.24-00319 PMC11744026

[17] Berg KM, Bray JE, Ng K-C, Liley HG, Greif R, Carlson JN, et al. 2023 international consensus on cardiopulmonary resuscitation and emergency cardiovascular care science with treatment recommendations: summary from the basic life support; advanced life support; pediatric life support; neonatal life support; education, implementation, and teams; and first aid task forces. Circulation. 2023;148(24):e187–280. 37942682 10.1161/CIR.0000000000001179 PMC10713008

[18] Sawyer KN, Camp-Rogers TR, Kotini-Shah P, Del Rios M, Gossip MR, Moitra VK, et al. Sudden Cardiac Arrest Survivorship: A Scientific Statement From the American Heart Association. Circulation. 2020;141(12):e654–85. 32078390 10.1161/CIR.0000000000000747

[19] Berg KM, Bray JE, Ng KC, Liley HG, Greif R, Carlson JN, et al. 2023 International consensus on cardiopulmonary resuscitation and emergency cardiovascular care science with treatment recommendations: summary from the basic life support; Advanced life support; Pediatric life support; Neonatal life support, education, implementation, and teams; and first aid task forces. Circulation. 2023;148(24):e187–280. 10.1161/CIR.0000000000001179 PMC10713008 37942682

[20] Tjelmeland IB, Wnent J, Masterson S, Kramer-Johansen J, Ong MEH, Smith K, et al. Did lockdown influence bystanders’ willingness to perform cardiopulmonary resuscitation? A worldwide registry-based perspective. Resuscitation. 2023;186:109764. 36934834 10.1016/j.resuscitation.2023.109764

[21] Goniewicz M, Bednarz K, Al-Wathinani AM, Goniewicz K. Assessment of Emergency Medical Service (EMS) response times and operational factors in out-of-hospital cardiac arrests (OHCA): a retrospective analysis. Adv Interventional Cardiology/Postępy w Kardiologii Interwencyjnej. 2024;20(1). 10.5114/aic.2024.145345 PMC11963042 40182110

